# Previous antiretroviral drug use compromises standard first-line HIV therapy and is mediated through drug-resistance

**DOI:** 10.1038/s41598-018-33538-0

**Published:** 2018-10-25

**Authors:** Seth C. Inzaule, Cissy M. Kityo, Margaret Siwale, Alani Sulaimon Akanmu, Maureen Wellington, Marleen de Jager, Prudence Ive, Kishor Mandaliya, Wendy Stevens, T. Sonia Boender, Pascale Ondoa, Kim C. E. Sigaloff, Denise Naniche, Tobias F. Rinke de Wit, Raph L. Hamers

**Affiliations:** 1 Amsterdam Institute for Global Health and Development, and Department of Global Health, Amsterdam UMC, University of Amsterdam, Amsterdam, The Netherlands; 2 Joint Clinical Research Centre, Kampala, Uganda; 3 Lusaka Trust Hospital, Lusaka, Zambia; 4 Department of Haematology and Blood Transfusion, Lagos University Teaching Hospital, and College of Medicine of the University of Lagos, Lagos, Nigeria; 5 Newlands Clinics, Harare, Zimbabwe; 6 Muelmed Hospital, Pretoria, South Africa; 7 Clinical HIV Research Unit, University of the Witwatersrand, Johannesburg, South Africa; 8 Coast Province General Hospital, Mombasa, Kenya; 9 Department of Molecular Medicine and Haematology, University of the Witwatersrand, Johannesburg and the National Health Laboratory Service, Johannesburg, South Africa; 10 Stichting HIV Monitoring, Amsterdam, The Netherlands; 11 African Society of Laboratory Medicine, Addis Ababa, Ethiopia; 12 ISGlobal, Barcelona Institute for Global Health Hospital Clínic, Universitat de Barcelona, Barcelona, Spain; 13 Department of Internal Medicine, Division of Infectious Diseases, Amsterdam UMC, Amsterdam, The Netherlands

## Abstract

In ART programs in sub-Saharan Africa, a growing proportion of HIV-infected persons initiating first-line antiretroviral therapy (ART) have a history of prior antiretroviral drug use (PAU). We assessed the effect of PAU on the risk of pre-treatment drug resistance (PDR) and virological failure (VF) in a multicountry cohort of HIV-infected adults initiated on a standard non-nucleoside reverse transcriptase inhibitor (NNRTI)-based first-line ART. Multivariate logistic regression was used to assess the associations between PAU, PDR and VF (defined as viral load ≥400 cps/mL). Causal mediation analysis was used to assess the proportion of the effect of PAU on VF that could be eliminated by intervening on PDR. Of 2737 participants, 122 (4.5%) had a history of PAU. Participants with PAU had a 7.2-fold (95% CI 4.4–11.7) risk of carrying PDR and a 3.1-fold (95% CI 1.6–6.1) increased risk of VF, compared to antiretroviral-naïve participants. Controlling for PDR would eliminate nearly half the effect of PAU on the risk of VF. Patients with a history of PAU are at increased risk of ART failure, which is to a large extent attributable to PDR. These findings support the recent WHO recommendations for use of differentiated, non-NNRTI-based empiric first-line therapy in patients with PAU.

## Introduction

In low and middle-income countries (LMICs), antiretroviral treatment (ART) regimens to treat HIV-1 infections are standardized under the WHO-defined public health approach^1^. Although reliable data are limited, ART programs in sub-Saharan Africa have reported that between 10 and 25% of first-line ART initiators have previously used antiretroviral drugs, either because they re-started ART after disengaging from care, or they used short-course antiretrovirals through prevention of mother-to-child transmission (PMTCT) programs, or pre- or post-exposure prophylaxis^2^.

People with previous antiretroviral drug use (denoted PAU) are at an increased risk of having drug-resistant HIV before starting ART (denoted pre-treatment drug resistance, PDR)^2,3^, which impairs response to standard non-nucleoside reverse transcriptase inhibitor (NNRTI)-based first-line ART^4–6^. However, for patients with PAU, few studies to date have evaluated the response to standard first-line ART or optimal management^7^. The vast majority of LMICs provide standard first-line therapy regardless of antiretroviral history or PDR testing^1,8^.

This study aimed to investigate the effects of PAU on PDR and virological failure (VF) in a multi-country cohort in sub-Saharan Africa, and the extent of which this effect could be eliminated by intervening on PDR.

## Methods

### Study design and population

The Pan-African Studies to Evaluate Resistance Monitoring (PASER-M) study was a prospective multi-country cohort including 13 sites in 6 countries (Kenya, Nigeria, South Africa, Uganda, Zambia, Zimbabwe), as profiled elsewhere^5^, conducted between 2007 and 2014. All participants were followed up according to local standard-of-care guidelines. The present study included all participants who initiated first-line ART containing an NNRTI plus two NRTIs. Retrospective viral load (VL) testing was performed before ART initiation and annually after ART initiation. Participants provided written informed consent at study enrolment. The study was approved by the appropriate research ethics committees at all collaborating sites and the Amsterdam UMC, University of Amsterdam, Institutional Review Board. The study was performed in accordance with relevant guidelines and regulations.

### Virological analysis

VL and PDR were retrospectively measured at either of two reference laboratories in Uganda and South Africa^5^. Sanger sequencing of the *pol* gene was performed if VL ≥ 1000 cps/ml using in-house assays. PDR was defined as the presence of ≥1 major drug resistance mutation (DRM) included in the International Antiviral Society–USA mutation list of December 2017 that are associated with any NRTI or the NNRTIs nevirapine or efavirenz^9^, plus the revertant mutations at codon 215 (A/C/D/E/N/S/V)^10^.

### Statistical analyses

#### Logistic regression analysis

Multivariate logistic regression with robust standard errors to account for clustering of observations within sites was used to assess the association between PAU and PDR and VF at month 12, defined as VL ≥ 400 cps/mL or a switch to second-line ART due to treatment failure up to 12 months. PAU was defined both as a dichotomous and a categorical variable according to type as follows: none, ART (standard triple ARV combinations), single-dose nevirapine (sdNVP) for PMTCT, or other ARV combinations (including mono/dual therapy). Models were adjusted for potential confounders, which were selected stepwise from the following list of independent variables: age, sex, country, calendar year of treatment initiation, type of NNRTI and NRTI, PDR, pretreatment VL and CD4 cell count, and the 12 months average of 30-day self-reported adherence. Subsequently, we investigated PDR as a potential effect modifier of the association between PAU and VF by including an interaction term in the model, and stratifying the model according to the presence/absence of PDR.

#### Causal mediation analysis

**Figure 1:**
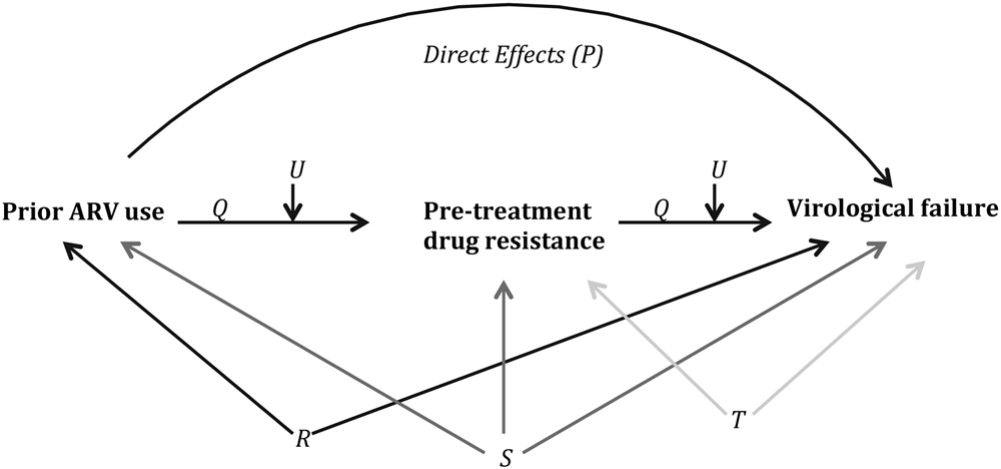
Direct acyclic graph (DAG) showing the relationship between prior ARV use, pre-treatment drug resistance and virological failure. P represents direct effects of prior ARV use on virological failure. Q represents indirect effects of prior ARV use on virological failure mediated through pre-treatment drug resistance. R represents confounders of the association between prior ARV use and virological failure: age and sex. S represents confounders of the association between pre-treatment drug resistance and virological failure that are affected by prior ARV use: pre-treatment viral load, CD4 cell counts and adherence. T represents confounders of the association between pre-treatment drug resistance and virological failure not included in S: type of initial antiretroviral treatment (NNRTI and NRTI) initiated and calendar year of treatment initiation. U represents unmeasured confounders. We note that the DAG is only a simple illustration and this may exclude complex relationships interacting between prior ARV use/pre-treatment drug resistance/virological failure and their confounders.

We also investigated PDR as a potential intermediate on the causal pathway of the association between PAU and VF using causal mediation analysis (Fig. 1)^11^. We calculated: 1) the *proportion mediated*, a measure that determines how much of the effect of the exposure (PAU) on the outcome (VF) is due to the effect of the exposure (PAU) on the intermediate (PDR). Proportion mediated is calculated as the ratio of natural indirect effect (NIE, effect of PDR on VF assuming all participants had PAU) to the total effect (TE), where *TE* = *NIE* + *NDE* (natural direct effects, effect of PAU on VF assuming PDR prevalence is similar in persons with/without PAU); 2) the *proportion eliminated*, a measure that determines the effect of the exposure (PAU) on the outcome (VF), that could be eliminated by intervening on the intermediate (PDR). Conceptually, this is the scenario where each patient receives a fully active ART regimen, either empirically or guided by PDR testing; therefore, by intervening on PDR we could eliminate a part of the effect of PAU on VF. Proportion eliminated is calculated as *TE-CDE*(*m* =0 )/*TE*, where CDE (controlled direct effects) is the effect of PAU on VF while fixing the intermediate PDR (m) to level 0. The causal mediation analysis was done using the paramed syntax in Stata with log-linear regression, assuming interaction^12^.

#### Sensitivity analyses

We performed the following sensitivity analyses to confirm the robustness of the associations: (1) We used a higher VL threshold of ≥ 1000 cps/mL to define VF (WHO definition)^1^; (2) We assessed the effect of PAU on acquired drug resistance (ADR) at 12 months for patients with VF ≥ 1000 cps/mL; (3) We further elucidated the effects of PDR on VF by restricting its definition to the presence of NNRTI-resistance (NNRTI-PDR); (4) We assessed the longer-term effect of PAU on VF (up to 24 months follow-up).

## Results

### Patient characteristics

Of the 2737 participants initiating ART, 122 (4.5%) had a documented history of PAU and 2615 (95.5%) were antiretroviral-naive; 23 (0.8%) participants were excluded because information on PAU was missing (Table 1). PAU comprised: ART (50%, 61/122), sdNVP (32%, 39/122), and other ARV combinations (18%, 22/122) (Table S1). Compared to antiretroviral-naïve participants, those with PAU were more likely to be female (5.9% vs 2.6%, p < 0.001), younger (median age 34.7 years [IQR 29.1–40.5] vs 37.0 [IQR 32.0–43.3], p < 0.001) with higher pre-treatment CD4 cell counts (median 177 [IQR 147–202] vs 133 [IQR 62–203] cells/μl, p = 0.0017) (Table 1). Females were more frequently exposed to sdNVP (2.6% vs 0%, p < 0.001) and other ARV combinations (1.3% vs 0.2%, p = 0.001), but not to ART (2.2% vs 2.4%, p = 0.813). The proportion of participants who had an average adherence level ≥95% did not differ between patients with (86.5%) or without (86.4%) PAU (p = 0.977).

**Table 1:** Baseline characteristics of patients with and without prior ARV use.

Characteristic	Prior ARV use N = 122	ARV naïve N = 2592	P-value
Age (years) Median IQR	34.7 (29.1–40.5)	37.0 (32.0–43.3)	<0.001
Sex, n (%)			
Female	29 (23.8)	1108 (42.8)	<0.001
Male	93 (76.2)	1484 (57.3)	
VL (log_10_) Median (IQR)	4.9 (4.1–5.6)	5.2 (4.4–5.6)	0.240
CD4 (Log_10_) Median (IQR)	177 (147–202)	133 (62–203)	0.0017
WHO clinical stage			0.275
I/II	54 (44.3)	1019 (39.3)	
III/IV	68 (55.7)	1573 (60.7)	
Type of initial NNRTI			
EFV	73 (59.8)	1545 (59.6)	0.964
NVP	49 (40.2)	1046 (40.4)	
Type of initial NRTI backbone			
TDF + XTC	43 (35.3)	866 (33.4)	0.790
d4T + 3TC	29 (23.8)	695 (26.8)	
ABC + 3TC	2 (1.6)	66 (2.6)	
ZDV + 3TC	48 (39.3)	964 (37.2)	
^†^ART adherence			0.977
≥95%	96 (86.5)	2044 (86.4)	
<95%	15 (13.5)	322 (13.6)	

Data are presented as n (%), unless stated otherwise.

3TC, lamivudine; ABC, abacavir; ART, antiretroviral therapy; d4T, stavudine; EFV, efavirenz; NVP, nevirapine; TDF, tenofovir; VL: viral load; XTC, lamivudine or emtricitabine; ZDV, zidovudine.

^†^Mean adherence measured as 30-day self-reported adherence over 12 months.

### Effect of PAU on PDR

**Figure 2:**
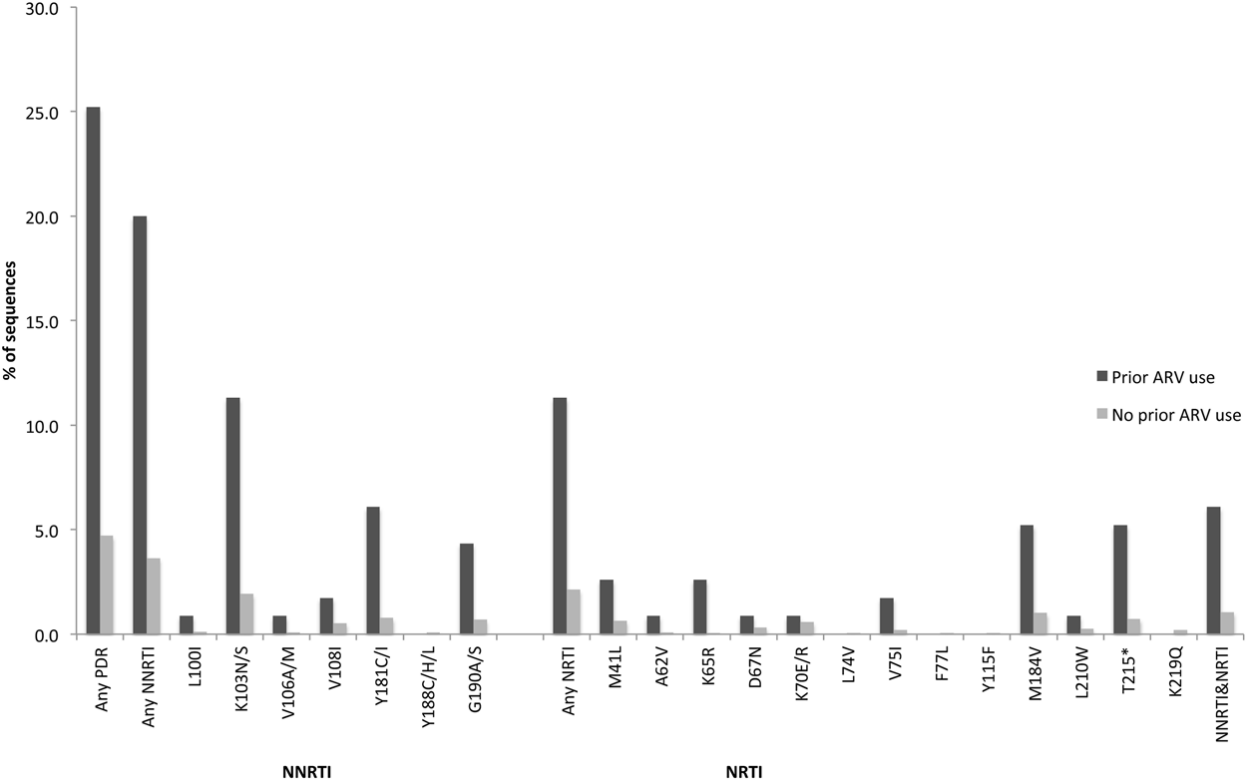
Patterns of drug resistance mutations in participants with and without prior ARV use Of all DRMs detected, 25.2% occurred in the participants with PAU. The proportion of participants who carried NNRTI, NRTI and dual NNRTI + NRTI resistance was 20.0%, 11.3% and 6.1% respectively for those with PAU and 3.6%, 2.1%, 1.1% for antiretroviral-naïve participants respectively.

2557/2714 (94.2%) participants had a PDR test performed, of whom 144 (5.6%) had PDR, with 115/2442 (4.7%) in antiretroviral-naïve participants and 29/115 (25.2%) among those with PAU (p < 0.001) (Fig. 2). The proportion of participants who carried any DRM, NNRTI-resistance, NRTI-resistance and dual-class resistance was: 29.1%, 27.3%, 12.7%, 10.9%, respectively, after ART; 28.6%, 14.3%, 19.1%, 4.8%, respectively, after other ARV combinations; 18.0%, 12.8%, 5.1%, 0.0%, respectively, after sdNVP; and 4.7%, 3.6%, 2.1%, 1.1%, respectively, for those who were antiretroviral-naive. In the adjusted analysis, the odds of PDR was 7.2-fold (95% CI 4.4–11.7; p < 0.001) higher in participants with PAU, compared to those who were antiretroviral-naive; and varied with the type of PAU: aOR 15.1 (95% CI, 5.3–42.5; p < 0.001) after other ARV combinations, 9.1 (95% CI 4.8–17.2; p < 0.001) after ART, and 3.3 (95% CI 1.4–8.1; p = 0.008) after sdNVP (Table 2).

**Table 2:** Associations between prior antiretroviral drug use, pre-treatment drug resistance and virological failure.

	N	Events	Unadjusted OR (95% CI)	P-value	Adjusted OR (95% CI)	P-value
***Effect of prior ARV use on PDR*** ^**a**^						
Any prior ARV use						
No	2534	288	1.0		1.0	
Yes	119	29	6.8 (4.3–10.8)	<0.001	7.2 (4.4–11.7)	<0.001
Type of prior ARV use						
None	2534	288	1.0		1.0	
ART	58	16	8.3 (4.5–15.3)	<0.001	9.1 (4.8–17.2)	<0.001
sdNVP	39	7	4.4 (1.9–10.2)	0.001	3.3 (1.4–8.1)	0.008
Other	22	6	8.1 (3.1–21.2)	<0.001	15.1 (5.3–42.5)	<0.001
***Effect of prior ARV use on VF*** ^**b**^						
Any prior ARV use						
No	1953	190	1.0		1.0	
Yes	86	20	2.7 (1.6–4.6)	<0.001	3.1 (1.6–6.1	0.001
Type of prior ARV use						
None	1953	190	1.0		1.0	
ART	4 4	13	3.6 (1.7–7.7)	0.001	3.9 (1.6–9.1)	0.002
sdNVP	29	5	1.9 (0.6–6.0)	0.256	3.1 (0.9–11.0)	0.088
Other	13	2	1.7 (0.6–4.7)	0.317	1.4 (0.5–4.0)	0.562
***Logistic regression model including interaction term*** (prior ARV use* PDR)^c^						0.485
***PDR as intermediate on causal pathway*** ^d^ ***Causal mediation analysis***						
Natural direct effects (NDE)			2.1 (1.2–3.8.)	0.013	2.7 (1.5–5.0)	0.002
Natural indirect effects (NIE)			1.4 (0.9–2.3)	0.1340	1.8 (1.0–3.1)	0.059
Controlled direct effect (CDE)			2.1 (1.1–4.1)	0.031	2.5 (1.2–5.1)	0.010
Total effects (TE)^e^			3.0(1.6–5.7)	0.001	4.8 (2.3–10.0)	<0.001
Proportion mediated PM = NIE/TE = 38%						
Proportion eliminated PE = (TE − CDE)/TE = 48%						

Abbreviations: ART, antiretroviral combination therapy; ARV, antiretroviral; PDR, pretreatment drug resistance; sdNVP, single-dose nevirapine for PMTCT; VF, virological failure’; NIE, natural indirect effects; NDE, natural direct effects; CDE, controlled direct effects; TE, total effects.

^a^Adjusted for age, sex, pre-treatment CD4 count, pre-treatment viral load, WHO clinical stage, calendar year of ART initiation, country.

^b^Adjusted for age, sex, pre-treatment CD4 count, pre-treatment drug resistance, type of ART, calendar year of ART initiation and adherence.

^c^Adjusted for age, sex, pre-treatment CD4 count, type of ART, calendar year of ART initiation and adherence.

^d^Adjusted for age, sex, pre-treatment CD4 counts, pre-treatment viral load, type of ART, calendar year of ART initiation and adherence.

^e^odds ratio for TE i.e. OR^TE^ = OR^NDE^*OR^NIE^.

### Effect of PAU on VF

#### Multivariable regression analysis

200/2031 (9.8%) participants who had a VL test result at 12 months experienced VF while 9 other patients had been switched to second-line treatment before 12 months. Of these 20/86 (23.3%) patients had PAU and 190/1953 (9.7%) were antiretroviral-naive (p ≤ 0.001). In the adjusted analysis, the odds of VF was 3.1-fold (95% CI 1.6–6.1; p = 0.001) higher in participants with PAU, compared to those who were antiretroviral-naive; and varied with the type of PAU: aOR 3.1 (95% CI 0.9–11.0; p = 0.088) after sdNVP, 3.9 (95% CI 1.6–9.1; p = 0.002) after ART, and 1.4 (95% CI 0.5–5.0; p = 0.562) after other ARV combinations (Table 2**)**.

The association between PAU and VF was similar when using a higher VL threshold (aOR 3.5, 95% CI 1.-7.4; p = 0.001) **(**sensitivity analysis 1; Table S2**)**. Genotypic results were available for 130/182 participants with a viral load >1000 cps/mL at 12 month, 95 (73%) of which had one or more major drug-resistance mutations. Compared with antiretroviral-naive participants, people with PAU had an increased risk of ADR (aOR 3.5, 95% CI 1.3–9.2; p = 0·010) (sensitivity analysis 2; Table S3).

#### Effect modification by PDR

In the regression model with interaction term, there was no evidence that PDR was an effect modifier of the association between PAU and VF (p for interaction = 0.485) (Table 2). Similarly, there was no evidence for interaction when we used a higher VL threshold (p for interaction = 0.432) (sensitivity analysis 1; Table S2) or when PDR was restricted to NNRTI-PDR (p for interaction = 0.451) (sensitivity analysis 3; Table S4).

#### Causal mediation by PDR

Table 1 summarizes the causal mediation analysis. The total effects of PAU on the risk of VF was aOR 4.8 (95% CI 2.3–10.0; p < 0.001). The proportion of the effect of PAU on VF mediated through PDR was 38%. The controlled direct effects of PAU, when fixing PDR = 0, remained statistically significantly associated with VF (aOR 2.7, 95% CI 1.5–5.0; p = 0.002). The proportion of the effect of PAU on VF that could be eliminated by intervening on PDR was 48%.

Compared to the main analysis, a VL threshold of >1000 cps/ml to define VF resulted in a slight reduction in the *proportion mediated* (33%) and an increase in the *proportion eliminated* (51%%) (sensitivity analysis 1; Table S2), and a restricted definition of NNRTI-PDR resulted in a similar *proportion mediated* (36%) and an increase in the *proportion eliminated* (63%) (sensitivity analysis 3; Table S4).

#### Long-term effect of PAU on VF (sensitivity analysis 4)

192/1838 (10%) participants who had a VL test result at 24 months experienced VF while 53 other patients had been switched to second-line treatment before 24 months. Of these 25/73 (34.2%) patients had PAU and 243/1818 (13.4%) were antiretroviral-naive (p ≤ 0.001). In the adjusted analysis, the odds of VF was 4.3-fold (95% CI 2.3–8.2; p < 0.001) higher in participants with PAU, compared to those antiretroviral-naive; and varied with the type of PAU: aOR 2.9 (95% CI 0.8–10.4; p = 0.110) after sdNVP, 6.7 (95% CI 3.0–14.6; p < 0.001) after ART, and 3.0 (95% CI 1.0–9.0; p = 0.055) after other ARV combinations (Table S5**)**.

Causal mediation analysis showed that the proportion of the effect of PAU on VF mediated through PDR was 24% and the proportion of the effect of PAU on VF that could be eliminated by intervening on PDR was 29%.

## Discussion

This prospective study among HIV-infected adults in sub-Saharan Africa starting first-line NNRTI-based ART found that persons who had a history of PAU, i.e. ART or sdNVP for PMTCT, were seven times more likely to have PDR, and three times more likely to experience VF within the first year of NNRTI-based ART, compared to those who were antiretroviral-naïve at ART initiation.

A causal mediation analysis provided two important additional insights. First, the pathway through the intermediate PDR was estimated to explain about 38% of the operation of the effect of PAU on VF. Conceptually, this means that the higher VF rates found in patients with PAU could partially be attributed to the presence of PDR, predominantly associated with the drug class of NNRTIs. We speculate that the residual effect of PAU could partially be attributed to unmeasured NNRTI-resistant minority variants, since the limited sensitivity of Sanger-based sequencing to detect minority virus populations may have resulted in an underestimation of the total effect of PDR. NNRTI-resistant minority variants have previously been shown to be associated with VF^13^, with higher impact among patients with PAU^14,15^.

Second, if we could eliminate the effect of the intermediate PDR on VF, the effect of PAU on VF is estimated to be reduced by half or more. Conceptually, this means that the use of an alternative fully-active first-line regimen in patients with PAU could half the number of failures that are attributable to PAU. This could be achieved by adopting either of two strategies in patients with PAU: the use of individualized PDR testing to guide the choice of first-line treatment, or a change of standard first-line regimen that is non-NNRTI-based (e.g. dolutegravir). The latter option has the advantage of addressing the potential residual impact of PAU on VF due to unmeasured minority resistant variants in absence of more sensitive resistance tests.

Previous studies on this topic are limited. Across seven WHO-led national surveys in LMICs, PDR was found to be considerably higher among persons with PAU (22%) than among antiretroviral-naive people (8%)^2^. A cross-sectional study in Nigeria found that patients with PAU were four times more likely to experience VF when initiated on NNRTI-based therapy^7^.

Our findings emphasize the importance of thorough assessment of previous antiretroviral history before ART initiation, and the use of non-NNRTI-based empiric first-line therapy (e.g. based on the integrase-inhibitor dolutegravir) in line with the latest WHO guidelines (July 2017)^8^. Our findings also provide further support to lifelong ART in childbearing women (PMTCT option B+) to avoid the risks associated with cycles of ART stopping and re-starting.

Our findings suggest that potential interventions to eliminate the effect of PDR (i.e. by providing an alternative fully-active first-line ART) could significantly reduce the risk of VF attributable to PAU. This impact is particularly substantive in reducing early VF during the first year of ART (48% risk reduction up to 12 months). However, in the longer term the impact may be more modest (29% risk reduction up to 24 months). These findings suggest that in the longer term the influence of PDR may be waning and that other factors explain the continuous impact of PAU on the risk of VF. We hypothesize that unaddressed factors associated with the initial default from care (hence the presence of PAU) may be undermining succesful adherence to long-term treatment, underscoring the need for enhanced adherence interventions for patients with PAU.

Strengths of the study were its prospective design, large sample, and the availibility of combined data on PAU, PDR and virological outcomes. The setting of routine ART programs enhanced the generalizibility of the results to other LMICs.

Study limitations were the lack of detailed PAU histories, precluding an in-depth analysis of attributes such as adherence, dosage, timing and duration, and the use of patient self-report and medical records to document PAU histories, with potential for recall and desirability bias^16^. This could have resulted in overall underestimation in the effect of PAU on VF. These limitations highlight the importance of enhancing electronic patient information systems that can link patient data across ART delivery sites.

In conclusion, patients with a history of PAU in African ART programs are at increased risk of ART failure, which is to a large extent attributable to the presence of PDR. To help meet the third of the UNAIDS global targets (i.e. ensuring viral suppression in 90% of people on ART), the choice of first-line ART regimens should be guided by a thorough assessment of antiretroviral history. Patients with PAU should receive differentiated, non-NNRTI-based empiric first-line therapy as recommended by the latest WHO guidelines.

### PASER-M collaborating sites

Lusaka Trust Hospital (M Siwale), Coptic Hospital (M Labib), KARA Clinic and Laboratory (J Menke), Lusaka, Zambia; Muelmed Hospital, Pretoria, South Africa (M E Botes, M de Jager); Themba Lethu Clinic, Clinical HIV Research Unit, (P Ive, and I Sanne) and Department of Molecular Medicine and Haematology (E Letsoalo, WS Stevens, K Steegen), University of the Witwatersrand, Johannesburg, South Africa; Acts Clinic, White River, South Africa (M Hardman); Newlands Clinic, Harare, Zimbabwe (M Wellington, R Luthy); Coast Province General Hospital, International Centre for Reproductive Health Kenya, Mombasa, Kenya (K Mandaliya); Mater Misericordiae Hospital, Nairobi, Kenya (M Dolan); Joint Clinical Research Centre, Fort Portal, Mbale and Kampala, Uganda (C Kityo, S Balinda, W Namala, H Namata, F Senono, R Nakanjako, M Mutebi, I Nankya, P Mugyenyi); Lagos University Teaching Hospital, Lagos, Nigeria (A Osibogun, S Akanmu, T Adeyemo, T Rodoye, H Adelabu); Department of Virology, University Medical Center, Utrecht, The Netherlands (R Schuurman); Amsterdam Institute for Global Health and Development, Kampala, Uganda (C Nalubwama, H Kakooza, M Nakitto, M O’Mello); Department of Global Health, Amsterdam UMC of the University of Amsterdam, Amsterdam Institute for Global Health and Development, Amsterdam, the Netherlands (RL Hamers, KCE Sigaloff, TS Boender, P Ondoa, N Pakker, FW Wit, JM Lange, TF Rinke de Wit).

## Electronic supplementary material

Supplementary tables

## Data Availability

All necessary data is included in the manuscript but any additional information is available upon request.
