# Supplementary material for: Previous antiretroviral drug use compromises standard first-line HIV therapy and is mediated through drug-resistance
Source: Sci Rep. 2018 Oct 25;8:15751. doi: 10.1038/s41598-018-33538-0 (PMC6202366; doi:10.1038/s41598-018-33538-0)
Supplement: Supplementary file 1 — Supplementary tables [file 41598_2018_33538_MOESM1_ESM.doc]

**Previous antiretroviral drug use compromises standard first-line HIV therapy and is mediated through drug-resistance**

**Authors**

Seth C. Inzaule^1^, Cissy M. Kityo^2^, Margaret Siwale^3^, Alani Sulaimon Akanmu^4^, Maureen Wellington^5^, Marleen de Jager^6^, Prudence Ive^7^, Kishor Mandaliya^8^, Wendy Stevens^9^, T.Sonia Boender^1,10^, Pascale Ondoa^1,11^, Kim C.E. Sigaloff¹, Denise Naniche^12^, Tobias F. Rinke de Wit^1^, Raph L. Hamers^1,13^

**Author affiliations**

1. Amsterdam Institute for Global Health and Development, and Department of Global Health, Academic Medical Center of the University of Amsterdam, Amsterdam, The Netherlands;
2. Joint Clinical Research Centre, Kampala, Uganda;
3. Lusaka Trust Hospital, Lusaka, Zambia;
4. Department of Haematology and Blood Transfusion, Lagos University Teaching Hospital, and College of Medicine of the University of Lagos, Lagos, Nigeria;
5. Newlands Clinics, Harare, Zimbabwe;
6. Muelmed Hospital, Pretoria, South Africa;
7. Clinical HIV Research Unit, University of the Witwatersrand, Johannesburg, South Africa
8. Coast Province General Hospital, Mombasa, Kenya
9. Department of Molecular Medicine and Haematology, University of the Witwatersrand, Johannesburg and the National Health Laboratory Service, Johannesburg, South Africa
10. Stichting HIV Monitoring, Amsterdam, The Netherlands;
11. African Society of Laboratory Medicine, Addis Ababa, Ethiopia.
12. ISGlobal, Barcelona Centre for International Health Research (CRESIB) Hospital Clínic, Universitat de Barcelona, Barcelona, Spain
13. Department of Internal Medicine, Division of Infectious Diseases, Academic Medical Center of the University of Amsterdam, and Amsterdam Infection and Immunity Institute, Amsterdam, The Netherlands;

| **Type of previous ARV use** | **N (%)** |
| --- | --- |
| *Single-dose NVP for PMTCT* | 39 (32) |
| *ART* | 61 (50) |
| d4T+3TC+NVP | 17 (28) |
| d4T+3TC+EFV | 10 (16) |
| ZDV+3TC+NVP | 8 (13) |
| ZDV+3TC+EFV | 5 (8) |
| TDF+FTC+EFV | 3 (5) |
| Unspecified | 18 (30) |
| *Other ARV combinations** | 22 (18%) |
| Dual-therapy PMTCT | 18 (82%) |
| Dual therapy non-PMTCT | 3(14%) |
| Mono-therapy^§^ | 1(5%) |

**Table S1: Details on types of antiretroviral regimens in patients with prior ARV use (N=122)**

3TC, lamivudine; ART, antiretroviral therapy; ARV, antiretroviral; d4T, stavudine; EFV, efavirenz; NVP, nevirapine; TDF, tenofovir; ZDV, zidovudine;

*Type of regimen was missing or not recorded;

^§^Included non-sdNVP for PMTCT

**Table S2: Effect of prior ARV use on virological failure,** defined by higher VL threshold (≥1000 cps/mL) (Sensitivity analysis #1)

|  | **N** | **Events** | **Unadjusted OR (95%CI)** | **P-value** | **Adjusted OR (95%CI)** | **P-value** |
| --- | --- | --- | --- | --- | --- | --- |
| ***Effect of prior ARV use on VF*** *^a^* | | | | | | |
| Any prior ARV use |  |  |  |  |  |  |
| No | 1953 | 163 | 1.0 |  | 1.0 |  |
| Yes | 86 | 19 | 2.9 (1.7-5.2) | <0.001 | 3.5 (1.7 -7.2) | 0.001 |
| ***Logistic regression model including interaction term*** (prior ARV use * PDR) ***^b^*** |  |  |  |  |  | 0.432 |
| ***Effect of prior ARV use on VF, PDR as intermediate on causal pathway ^c^*** | | | | | | |
| *Causal mediation analysis* | | | | | | |
| Natural direct effects (NDE) |  |  | 2.3 (1.2-4.2) | 0.008 | 3.1 (1.6-5.8) | 0.001 |
| Natural indirect effects (NIE) |  |  | 1.5 (0.9-2.4) | 0.109 | 1.9 (1.0-3.4) | 0.043 |
| Controlled direct effect (CDE) |  |  | 2.2 (1.1-4.5) | 0.008 | 2.8 (1.3-5.8) | 0.007 |
| Total effects (TE)^d^ |  |  | 3.4 (1.8-6.5) | <0.001 | 5.7 (2.6-12.4) | <0.001 |
| Proportion mediated PM = NIE / TE = 33% |  |  |  |  |  |  |
| Proportion eliminated PE = (TE – CDE) / TE =51% |  |  |  |  |  |  |

Abbreviations: ART, antiretroviral combination therapy; ARV, antiretroviral; PDR, pretreatment drug resistance; VF, virological failure; NIE, natural indirect effects; NDE, natural direct effects; CDE, controlled direct effects; TE, total effects

^a^ Adjusted for age, sex, pre-treatment CD4 counts, pre-treatment viral load, PDR, type of ART, calendar year of ART initiation and adherence

^b^ Adjusted for age, sex, pre-treatment CD4 counts, pre-treatment viral load, type of ART, calendar year of ART initiation and adherence

^c^ Adjusted for age, sex, pre-treatment CD4 counts, pre-treatment viral load, type of ART, calendar year of ART initiation and adherence

^d^ odds ratio for TE i.e. OR^TE^=OR^NDE^*OR^NIE^

**Table S3: Effect of prior ARV use on acquired drug resistance (Sensitivity analysis #2)**

|  | **N** | **Events** | **Unadjusted OR (95%CI)** | **P-value** | **Adjusted OR (95%CI)** | **P-value** |
| --- | --- | --- | --- | --- | --- | --- |
| ***Effect of prior ARV use on ADR*** *^a^* | | | | | | |
| Any prior ARV use |  |  |  |  |  |  |
| No | 1883 | 85 | 1.0 |  | 1.0 |  |
| Yes | 77 | 10 | 3.2 (1.6-6.4) | 0.001 | 3.5 (1.3 -9.0) | 0.011 |

Abbreviations: ADR, acquired drug resistance; ARV, antiretroviral

^a^ Adjusted for age, sex, pre-treatment CD4 counts, pre-treatment viral load, type of ART, calendar year of ART initiation and adherence

Analysis included 12 months viral load + acquired drug resistance as a composite outcome variable

**Table S4: Associations between prior antiretroviral drug use, pre-treatment NNRTI drug resistance and virological failure (Sensitivity analysis #3)**

|  | **N** | **Events** | **Unadjusted OR (95%CI)** | **P-value** | **Adjusted OR (95%CI)** | **P-value** |
| --- | --- | --- | --- | --- | --- | --- |
| ***Logistic regression model including interaction term*** *(prior ARV use * NNRTI PDR**^b^* |  |  |  |  |  | 0.451 |
| ***Effect of prior ARV use on VF, NNRTI-PDR as intermediate on causal pathway ^c^*** | | | | | | |
| Natural direct effects (NDE) |  |  | 2.1 (1.2-3.7) | 0.015 | 2.8 (1.5-5.2) | 0.001 |
| Natural indirect effects (NIE) |  |  | 1.7(1.0-2.9) | 0.066 | 2.3 (1.1-4.4) | 0.019 |
| Controlled direct effect (CDE) |  |  | 2.0 (1.0-3.8) | 0.048 | 2.4 (1.2-4.9) | 0.014 |
| Total effects (TE)^d^ |  |  | 3.5 (1.7-7.3) | <0.001 | 6.4 (2.5-16.1) | <0.001 |
| Proportion mediated PM = NIE / TE =36% |  |  |  |  |  |  |
| Proportion eliminated PE = TE – CDE / TE =63% |  |  |  |  |  |  |

Abbreviations: ART, antiretroviral combination therapy; ARV, antiretroviral; NNRTI, non-nucleoside reverse transcriptase inhibitor; PDR, pretreatment drug resistance; VF, virological failure, NIE, natural indirect effects; NDE, natural direct effects; CDE, controlled direct effects; TE, total effects;

^a^ Adjusted for age, sex, pre-treatment CD4 count, pre-treatment viral load, country, WHO clinical stage, calendar year of ART initiation

^b^ Adjusted for age, sex, pre-treatment CD4 count, pre-treatment drug resistance, type of ART, calendar year of ART initiation and adherence

^c^ Adjusted for age, sex, pre-treatment CD4 count, pre-treatment viral load, type of ART, calendar year of ART initiation and adherence

^d^ Odds ratio for TE i.e. OR^TE^=OR^NDE^*OR^NIE^

**Table S5: Effect of prior ARV use on virological failure, up to 24 months follow-up (Sensitivity analysis #4**)

|  | **N** | **Events** | **Unadjusted OR (95%CI)** | **P-value** | **Adjusted OR (95%CI)** | **P-value** |
| --- | --- | --- | --- | --- | --- | --- |
| ***Effect of prior ARV use on VF*** *^a^* | | | | | | |
| Any prior ARV use |  |  |  |  |  |  |
| No | 1818 | 243 | 1.0 |  | 1.0 |  |
| Yes | 73 | 25 | 3.2 (2.0-5.3) | <0.001 | 4.3 (2.3 -8.2) | <0.001 |
| Type of prior ARV use |  |  |  |  |  |  |
| None | 1818 | 243 | 1.0 |  | 1.0 |  |
| ART | 36 | 16 | 4.9 (2.7-8.8) | <0.001 | 6.7(3.0-14.6) | <0.001 |
| sdNVP | 26 | 6 | 1.9 (0.6-6.1) | 0.259 | 2.9 (0.8-10.4) | 0.110 |
| Other | 11 | 3 | 2.4 (0.8-7.1) | 0.107 | 3.0 (1.0-9.0) | 0.055 |
| ***Logistic regression model including interaction term*** (prior ARV use * PDR) ***^b^*** |  |  |  |  |  | 0.767 |
| ***Effect of prior ARV use on VF, PDR as intermediate on causal pathway ^c^*** | | | | | | |
| *Causal mediation analysis* | | | | | | |
| Natural direct effects (NDE) |  |  | 3.0 (1.7-5.1) | <0.001 | 3.8 (2.1-6.9) | <0.001 |
| Natural indirect effects (NIE) |  |  | 1.3 (0.9-2.0) | 0.223 | 1.4(0.9-2.4) | 0.164 |
| Controlled direct effect (CDE) |  |  | 3.1 (1.7-5.6) | <0.001 | 3.9(2.0-7.4) | <0.001 |
| Total effects (TE)^d^ |  |  | 3.8 (2.1-7.0) | <0.001 | 5.5 (2.8-11.0) | <0.001 |
| Proportion mediated PM = NIE / TE = 24% |  |  |  |  |  |  |
| Proportion eliminated PE = (TE – CDE) / TE = 29% |  |  |  |  |  |  |

Abbreviations: ART, antiretroviral combination therapy; ARV, antiretroviral; PDR, pretreatment drug resistance; VF, virological failure; NIE, natural indirect effects; NDE, natural direct effects; CDE, controlled direct effects; TE, total effects

^a^ Adjusted for age, sex, pre-treatment CD4 counts, pre-treatment viral load, PDR, type of ART, calendar year of ART initiation and adherence

^b^ Adjusted for age, sex, pre-treatment CD4 counts, pre-treatment viral load, type of ART, calendar year of ART initiation and adherence

^c^ Adjusted for age, sex, pre-treatment CD4 counts, pre-treatment viral load, type of ART, calendar year of ART initiation and adherence

^d^ Odds ratio for TE i.e. OR^TE^=OR^NDE^*OR^NIE^
